# Accuracy Assessment of the Integration of GNSS and a MEMS IMU in a Terrestrial Platform

**DOI:** 10.3390/s141120866

**Published:** 2014-11-04

**Authors:** Sergio Madeira, Wenlin Yan, Luísa Bastos, José A. Gonçalves

**Affiliations:** 1 University of Trás-os-Montes e Alto Douro, Vila Real 5000-801, Portugal; INESC TEC - Technology and Science, Rua Dr. Roberto Frias, 4200-465 Porto, Portugal; 2 Department of Geosciences Environment and Spatial Planning, Faculty of Science, University of Porto, Rua Campo Alegre 687, Porto 4169-007, Portugal; E-Mails: williamtown.yan@gmail.com (W.Y.); lcbastos@fc.up.pt (L.B.); jagoncal@fc.up.pt (J.A.G.); 3 CIIMAR/CIMAR, University of Porto, Rua dos Bragas 289, Porto 4050-123, Portugal

**Keywords:** Mobile Mapping System, Global Navigation Satellite Systems, Inertial Navigation System, GNSS/IMU

## Abstract

MEMS Inertial Measurement Units are available at low cost and can replace expensive units in mobile mapping platforms which need direct georeferencing. This is done through the integration with GNSS measurements in order to achieve a continuous positioning solution and to obtain orientation angles. This paper presents the results of the assessment of the accuracy of a system that integrates GNSS and a MEMS IMU in a terrestrial platform. We describe the methodology used and the tests realized where the accuracy of the positions and orientation parameters were assessed using an independent photogrammetric technique employing cameras that integrate the mobile mapping system developed by the authors. Results for the accuracy of attitude angles and coordinates show that accuracies better than a decimeter in positions, and under a degree in angles, can be achieved even considering that the terrestrial platform is operating in less than favorable environments.

## 1. Introduction

A Direct Georeferencing System (DGS) can be defined as a set of sensors onboard a platform, whose goal is to obtain positions (as three coordinates) and attitudes (as three angles) of the origin of a reference system, defined in the platform, without external control points. One main application of a DGS is as a major component of a Mobile Mapping System (MMS), which contains, besides the DGS, a set of remote sensors to acquire information from the surrounding objects. The linkage of absolute positions and orientation parameters to the remote sensors allows the determination of positional and geometrical information of the objects observed [1]. Detailed descriptions of this technology can be found for example in [2,3].

In terms of applications, terrestrial MMS can be used to acquire data of urban or road infrastructures and can be optimized for specific applications such as traffic sign inventory and railway or road inspection as described in [4–6]. More specific applications of terrestrial MMS are the automatic DTM (Digital Terrain Model) generation in sandy beaches [7], river shorelines change detection [8] or pavement surface analysis [9].

Nowadays two lines emerge in MMS. A first one associated with high to moderate cost navigation or tactical grade Inertial Measuring Units (IMUs) and laser/camera sensors, which when integrated with satellite positioning receivers (Global Navigation Satellite Systems—GNSS) can deliver high accuracy surveys/modeling. The level of accuracy achieved by GNSS/IMU systems used in DGS can vary significantly, depending on the grade of the IMUs and on the GNSS data used. For a tactical grade IMU, integrated with geodetic grade GNSS receivers (multi-frequency code and phase receivers), accuracies can be of the order of one decimeter for positions, and better than 0.03° for attitude [10]. Navigation grade IMUs can provide accuracies of one order of magnitude better. Some of these types of systems are commercially available and are mainly directed to road, urban or railway environment surveys. Low cost Micro Electromechanical System (MEMS) IMUs integrated with geodetic grade GNSS receivers can provide results at the several decimeter level for positions and 0.2° for attitude, as shown for example in [11,12]. A very robust way of developing a DGS is by integrating GNSS and IMU measurements—an insight into this approach can be found in [13,14], or [15]. Manufacturers of GNSS receivers and IMUs provide data sheets with information on the expected accuracy that these individual systems can achieve, which usually relates to optimal operating conditions. However, in terrestrial platforms the working environment is more aggressive and consequently an accuracy decrease can be expected. In a MMS, the accuracy performance of the DGS is critical to the final positional accuracy of the objects acquired by the data sensors.

The subject of accuracy evaluation or calibration of MEMS units using known orientation and positions was presented by Artese *et al.* [16]. El-Sheimy presented a standard technique to obtain relative orientation between pairs of imaging sensors and DGS by using external control points on a bundle adjustment with known parameters as constraints [17].

The main goal of the work presented here was to assess the linear and angular accuracy of a DGS mounted on a terrestrial platform previously developed by the authors and described in [7], considering good conditions for GNSS observation. The tested DGS is composed by a dual frequency GNSS receiver and a MEMS IMU. The methodology used for the accuracy assessment is based on the comparison of the parameters given by the DGS with those obtained independently by photogrammetric techniques, using control points.

In Section 2, we describe briefly the DGS used and address the problem of converting data between different reference systems (sensors and platform), including the used conventions. In Section 3 the surveying test carried out is also described, as well as the methodology to assess the accuracy of the DGS system implemented. Finally, in Section 4, a brief discussion of the results obtained is presented.

## 2. Equipment and Methodology

The implemented DGS relies on the integration of measurements from a GNSS phase receiver with measurements from an IMU through a Kalman filter, which is a classic configuration that can provide very high accuracy due to its complementarity. A description of GNSS and IMU systems is not in the scope of this work since there is enough and accessible literature on the subjects (for GNSS see for example [18] and for IMU [19]).

### 2.1. Equipment Used

The accuracy of a GNSS/IMU system depends on the equipment used, built in processing and integration software. The DGS equipment used in our case was a Novatel dual phase GNSS receiver (Novatel Inc., Calgary, Canada) and a AHRS440 IMU from Crossbow (Crossbow: San Jose, CA, USA). Tables 1 and 2 present their accuracies, taken from the manufacturers' specifications.

The remote sensors used were two digital CCD cameras whose characteristics are presented in Table 3.

**Figure 1.**
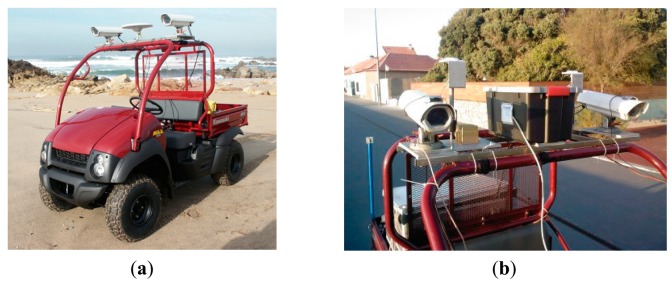
(**a**) Surveying vehicle; (**b**) Equipment detail.

The cameras were calibrated independently with a methodology developed by the authors [20]. The resulting five parameters (principal point coordinates, focal distance and two radial distortions) allows for a mean linear re-projection error of 0.35 pixels. The calibrated focal distances, always fixed in the maximum value, were approximately 3.6 mm for both cameras. Images of the vehicle and the equipment mounted on it can be viewed in Figure 1.

### 2.2. GNSS/INS Integration by Kalman Filter

The Least Squares method is the most common way to estimate the state vector in geomatics, which is purely based on measurements [21]. For navigation applications it is more efficient to use a sequential approach and here the Kalman Filter plays an important role in the integration of GNSS and IMU observations [22–25], which is based on the knowledge of the measurements and the state vector dynamics.

In the Kalman Filter mechanical equations, the position, velocity and attitude computed in the navigation frame, combined with other information, such as sensors errors and local gravity disturbance, are taken as the states of the filter. Here we have used a 34 state vector for the Extend Kalman Filter (EKF) described as:

(1) $${\begin{array}{lllllllllllllll} \text{x}= & [ & {\text{r}}_{e}^{l} & {\text{v}}_{e}^{1} & {\text{C}}_{b}^{l} & {\text{b}}_{a} & {\text{b}}_{\omega } & {\text{s}}_{a} & {\text{s}}_{\omega } & \text{s}{\text{a}}_{a} & \text{s}{\text{a}}_{w} & \delta_{a} & \delta_{\omega } & \delta g & ] \end{array}}^{\text{T}}$$

where the thirty four states comprise: three position coordinates ${\text{r}}_{e}^{l}$, three velocities ${\text{v}}_{e}^{l}$, three attitude angles (expressed by direction cosine matrix $C_{b}^{l}$), six constant bias ***b****_a_* and ***b****_ω_*, six scale bias ***s****_a_* and ***s****_ω_*, six scale asymmetry ***sa****_a_* and ***sa****_w_*, six misalignment parameters *δ_a_* and *δ_ω_* and one gravity disturbance *δ_g_*. The related error states are:

(2) $$\delta \text{x}={[\delta {\text{r}}_{e}^{l}\delta {\text{v}}_{e}^{l}\psi^{l}\varepsilon {\text{b}}_{a}\varepsilon {\text{b}}_{\omega }\varepsilon {\text{s}}_{a}\varepsilon {\text{s}}_{\omega }\varepsilon \text{s}{\text{a}}_{a}\varepsilon \text{s}{\text{a}}_{\omega }\varepsilon \delta_{a}\varepsilon \delta_{\omega }\varepsilon \delta g]}^{\text{T}}$$

The dynamic error equation is:

(3) $$\delta \dot{\text{x}}=\text{F}\delta \text{x}+\text{Gw}$$

where **F**_34×34_ is the system dynamics matrix; **G**_34×7_ is the disturbance input matrix; ***w***_7×7_ is the system white noise matrix. A typical Kalman Filter dynamic equation in our application can be written as:

(4) $${\text{x}}_{k}^{-}=\Phi_{k-1}{\text{x}}_{k-1}$$

(5) $${\text{P}}_{k}^{-}=\Phi_{k-1}{\text{P}}_{k-1}\Phi_{k-1}^{T}+{\text{Q}}_{k-1}$$

where the superscript “-” means “a prediction estimation”; the subscript “k” is the current index of discrete samplings; **P**_34×34_ is the covariance matrix of the state vector; **Φ***_k_*_−1_ is the state transition matrix, describing how the system changes over time, which can be approximated by the equation:

(6) $$\Phi_{k-1}\approx \text{I}+{\text{F}}_{k-1}\Delta t+\frac{1}{2!}{\left({\text{F}}_{k-1}\Delta t\right)}^{2}+\frac{1}{3!}{\left({\text{F}}_{k-1}\Delta t\right)}^{3}$$

The noise matrix **Q***_k_*_−1_ can be approximated as:

(7) $${\text{Q}}_{k-1}\approx {\text{G}}_{k-1}\text{Q}\Delta t{\text{G}}_{k-1}^{T}$$

where ***Q*** is the so called Spectral Density Matrix, which is a function of white noise ***w*** [26]:

(8) $$\text{Q}(t)\delta (t-\tau )=E[\text{w}(t)\text{w}{\left(t\right)}^{T}]$$

In this equation*t* is the current observation time, *τ* means the time difference between consecutive observations and *δ* is the Dirac delta function with dimension s^−1^; In our application the ***Q****_k_*_−1_ has the form:

(9) $${\text{Q}}_{k-1}=\text{diag}{\left(\begin{array}{ccccccc} \sigma_{ax}^{2} & \sigma_{ay}^{2} & \sigma_{az}^{2} & \sigma_{wx}^{2} & \sigma_{wy}^{2} & \sigma_{wz}^{2} & \sigma_{\delta g}^{2} \end{array}\right)}_{7\times 7}$$

where **σ***_a_* and **σ***_w_* are the standard deviations of the accelerometers and gyros; **σ***_δg_* is the standard deviation of the gravity. In high precision demanding applications, **Q** must be specified carefully, particularly for the low cost IMU. If **Q** is set too small, the Kalman Filter will trust the measurements more than the dynamic equation. In the EKF a typical representation for **Q** can be given as:

(10) $${\text{Q}}_{d}^{k-1}\approx \frac{1}{2}[\Phi_{k-1}{\text{G}}_{k-1}{\text{Q}}_{k-1}{\text{G}}_{k-1}^{T}\Phi_{k-1}^{T}+{\text{G}}_{k}{\text{Q}}_{k}{\text{G}}_{k}^{T}]\Delta t$$

The system observation equations can be written as:

(11) $${\text{z}}_{k}={\left[\begin{array}{c} {\text{r}}_{\text{imu}}^{l}-{\text{r}}_{\text{gnss}}^{l}\\ {\text{v}}_{\text{imu}}^{l}-{\text{v}}_{\text{gnss}}^{l} \end{array}\right]}_{6\times 1}={\text{H}}_{k}{\text{x}}_{k}^{-}+{\text{v}}_{k}$$

where **H***_k_* is the measurement matrix and ***v****_k_* is a zero mean Gaussian noise. When the GNSS updates are available the update process can be done using the following equations:

(12) $${\text{K}}_{k}={\text{P}}_{k-1}^{-}{\text{H}}_{k}^{T}{\left[{\text{H}}_{k}{\text{P}}_{k-1}^{-}{\text{H}}_{k}^{T}+{\text{R}}_{k}\right]}^{-1}$$

(13) $${\text{x}}_{k}^{+}={\text{x}}_{k}^{-}+{\text{K}}_{\text{k}}[{\text{z}}_{k}-{\text{H}}_{k}{\text{x}}_{k}^{-}]$$

(14) $${\text{P}}_{k}^{+}=[\text{I}-{\text{K}}_{k}{\text{H}}_{k}]{\text{P}}_{k-1}^{-}$$

where the superscript “+” means “a corrected estimation”; **K***_k_* is the so-called Kalman gain; **R***_k_* is the measurement noise covariance matrix.

### 2.3. Coordinate Transformation between Reference Systems

**Figure 2.**
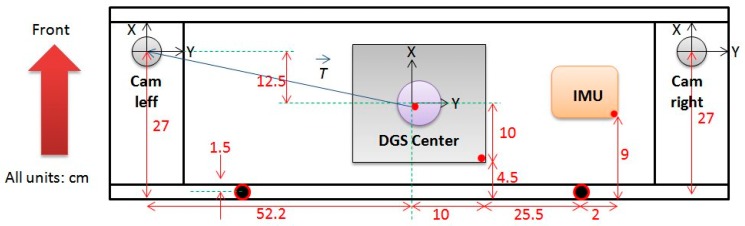
Platform plan with components location and measured lengths.

Figure 2 shows a plan of the platform used to carry out the surveys. Complementary information about the plan is that the cameras are leveled over the platform, as can be viewed in Figure 1b, and their centers are two centimeters above the center of the DGS. The lengths between components were measured rigorously and the values are shown. There are four reference systems (r.s.) present: the Cam left, Cam right, DGS (whose origin is considered at the center of the platform) and the cartographic r.s. in which the platform moves. In order to convert coordinates of points between reference frames, three linear and three angular components are needed.

Considering any of these r.s., say 1 and 2, assuming that only scale is maintained, then the following equation can be established relating to a point in space (*P*) [27]:

(15) $${\left[X\right]}_{1}={M_{21}}^{-1}.{\left[X\right]}_{2}+{\left[X0_{2}\right]}_{1}$$

where [*X*]_1_ contains the coordinates of *P* in 1st r.s., [*X*]_2_ the coordinates of *P* in 2nd r.s., [*X0*]_1_ contains the coordinates of the origin of the 2nd r.s. in the 1^st^ r.s. and *M*_21_ is a matrix that explains the rotation from the 1st to the 2nd r.s.

Considering the observation of the rotation angles, and the positions of cameras and *DGS* in the cartographic r.s., the following equations can be established using Equation (15):

(16) $${\left[X\right]}_{\text{Cart}}={M_{\text{LcamCart}}}^{-1}\cdot {\left[X\right]}_{\text{Lcam}}+{\left[X0_{\text{Left}}\right]}_{\text{Cart}}$$

(17) $${\left[X\right]}_{\text{Cart}}={M_{\text{RcamCart}}}^{-1}\cdot {\left[X\right]}_{\text{Rcam}}+{\left[X0_{\text{Rcam}}\right]}_{\text{Cart}}$$

(18) $${\left[X\right]}_{\text{Lcam}}={M_{\text{RcamLCam}}}^{-1}\cdot {\left[X\right]}_{\text{Rcam}}+{\left[X0_{\text{Rcam}}\right]}_{\text{Lcam}}$$

(19) $${\left[X\right]}_{\text{Cart}}={M_{\text{DGSCart}}}^{-1}\cdot {\left[X\right]}_{\text{DGS}}+{\left[X0_{\text{DGS}}\right]}_{\text{Cart}}$$

(20) $${\left[X\right]}_{\text{DGS}}={M_{\text{LcamDGS}}}^{-1}\cdot {\left[X\right]}_{\text{Lcam}}+{\left[X0_{\text{Left}}\right]}_{\text{DGS}}$$

where the *M* matrices contain the rotation angles in the indicated directions and the *X*0 contains the cartographic coordinates (X, Y, H) of the origins of the r. s. Using Equations (16)–(18) the relative orientation parameters of the right camera relatively to the left camera can be deduced:

(21) $$M_{\text{RcamLcam}}=M_{\text{RcamCart}\cdot }{M_{\text{LcamCart}}}^{-1}$$

(22) $${\left[X0_{\text{Rcam}}\right]}_{\text{Lcam}}=M_{\text{LcamCart}}.\left({\left[X0_{\text{Rcam}}-\text{X}0_{\text{Lcam}}\right]}_{\text{Cart}}\right)$$

Using Equations (16), (19) and (20) the offset parameters relating the DGS with the left camera are:

(23) $$M_{\text{LcamDGS}}=M_{\text{LcamCart}}.{M_{\text{DGSCart}}}^{-1}$$

(24) $${\left[X0_{\text{Lcam}}\right]}_{\text{DGS}}=M_{\text{DGSCart}}.\left({\left[X0_{\text{Lcam}}-X0_{\text{DGS}}\right]}_{\text{Cart}}\right)$$

These results will be used as needed in the rest of the document.

### 2.4. Rotation Angles (*ω φ κ* versus Roll, Pitch, Heading)

Traditionally, in photogrammetry, the orientation angles were associated with aerial photography and used to calculate Earth referenced coordinates from the photographic coordinates. They are the well-known rotations *ω ϕ κ*, of the photographic camera axes [27].

With the development of navigation technology applied to mobile surveying platforms, orientation angles different from the usual ones in photogrammetry were adopted, namely the roll, pitch and heading angles—see Table 4.

In the case of the terrestrial image, the authors used an axis convention different from the usual in photogrammetry, namely by considering photo coordinates as (*x,z*) and the *y* axis forward from the photo, in so achieving a more intuitive relation between *κ* and *heading* angles. Despite the axes considered in each case being different and rotated in different order, the resulting rotation matrices are the same [27]. In this work the conventions used are presented in Table 4. In each case the rotation angles can be obtained from the rotation matrices as follows:

(25) $$\begin{array}{l} \omega ,\phi ,\kappa \;\text{case}\begin{array}{cc} \begin{array}{cc} : & \omega =\arctan\left(-\frac{{\text{r}}_{32}}{{\text{r}}_{33}}\right) \end{array} & \begin{array}{cc} \varphi =\arcsin\left(r_{31}\right) & \kappa =\arctan\left(-\frac{r_{21}}{r_{11}}\right) \end{array} \end{array} \end{array}$$

(26) $$\begin{array}{ll} \begin{array}{cc} \text{roll},\text{pitch},\text{heading}\;\text{case}: & \text{roll}=\arctan\left(-\frac{r_{13}}{r_{33}}\right) \end{array} & \begin{array}{cc} \text{pitch}=\arcsin({\text{r}}_{23}) & \text{heading}=\arctan\left(-\frac{r_{21}}{r_{22}}\right) \end{array} \end{array}$$

where *r_ij_* represents (i,j) matrix elements.

## 3. Evaluation of the DGS Accuracy

In order to evaluate the quality of the obtained navigation solution, the authors compared the exterior orientation parameters obtained independently from the cameras on the platform, with the parameters derived from the DGS itself. The offsets between the DGS and the cameras must be considered. However only the linear offsets are known by means of rigorous measurements over the platform (Figure 2). In fact, offset determination using the independent observations from cameras and DGS were used in this work to estimate the accuracy of the later. The observations, the strategy adopted and the results obtained are described below.

### 3.1. Observations

The data acquisition was conducted in an urban environment with the terrestrial video-based MMS, described in [7]. The test zone was selected on pavement with painted crosswalks because there we have well-defined points with enough variability on the ground and in the photos and also because such observations may be easily repeated at other locations in order to apply this methodology in our future work.

**Figure 3.**
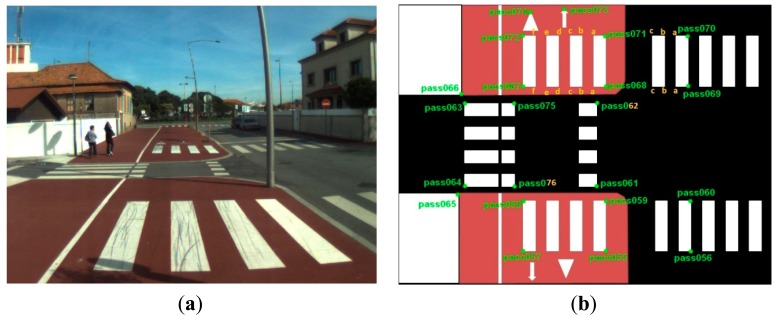
Test zone of the terrestrial case: (**a**) general view; (**b**) scheme of the observed control.

The control points were observed with centimetric accuracy by means of a static relative GNSS survey (referring Table 1, the acquisition mode was RT-2 (Real Time Kinematics), with the reference station at a distance of 6 km. Figure 3 shows a general view of the test area (a) and a scheme with the observed control points (b).

Several passes were made through the test zone, from different directions, either following a straight line or a curve, collecting images at a rate of four per second (the cameras collect images at the same instant since they share the same trigger). Of these, nine pairs were selected, in which a sufficient number of control points could be observed, in order to compare the position and orientation parameters with those given by the DGS. This means that the tests were carried using eighteen images in which the control points could be observed. Of interest is the fact that the 2nd and 3rd pairs were selected because the vehicle was moving in a curve, and all the other were along a straight trajectory.

### 3.2. Position and Orientation Parameters by Space Resection and Respective Accuracy Estimation

The position and orientation parameters of the cameras in the cartographic space were obtained by space resection from the control points. This is a photogrammetric process by which the six exterior orientation parameters of an image are computed using at least 3 control points on the photo [27]. The image coordinates of the control points were identified manually and independently in each photo. The results of this phase are summarized in Table 5.

Table 5 contains the images used to perform exterior orientation, including the used control points, the exterior orientation parameters obtained and, in the right column, the calculated relative orientation parameters. The relative orientation matrices and linear relative parameters were obtained with Formulas (21) and (22) and the relative orientation angles were extracted from the rotation matrices as in Formula (25).

The relative orientation between cameras is constant for all pairs because they are fixed on the platform and the images of each pair were obtained at the same instants. Moreover, the exterior orientation of the images, as referred previously, was obtained independently in each photo. In this way it is expected that the relative orientation parameters calculated, regarded as observations, follow a normal law, so their standard deviations are a good estimate of the accuracy of this methodology for obtaining the exterior orientation of an image. Table 6 presents the accuracy estimations in the orientation angles, in the 3D positions and in the length of vector *T*.

The length separation between the two camera centers is known to be 1.044 m (as shown in Figure 2). In this case it is possible to present the *root mean square error* and the *mean error* of the length of vector *T*, what is also done in Table 6. This is a good estimate of the accuracy achieved by space resection, for the positional parameters.

### 3.3. Position and Orientation Parameters Given by the GNSS / IMU System and Respective Accuracy Estimation

After processing the IMU and GNSS observations, as described in Section 2.2, a navigation solution was obtained for the surveyed path. Of interest are the instants that correspond to the analyzed photogrammetric pairs. The results obtained are presented in Table 7.

Estimation of the accuracy of the IMU, either in coordinates or orientations, cannot rely on the differences between IMU and cameras observations as these depend on the directions of the vehicle. The followed strategy was to calculate the linear and angular offsets of the left camera in the IMU r.s., for each observation, which, in the absence of observation errors, should be constant.

The offset matrices, *M_LcamDGS_*, were calculated using Formula (23), where *M_LcamCart_* is the left camera rotation matrix, calculated with the rotation angles in Table 5, and *M_DGSCart_* is the rotation matrix of DGS calculated with the rotation angles in Table 7. The offset angles where extracted using Formula (26). The linear offsets were calculated using Formula (24), where [*X*0*_Lcam_* − *X*0*_DGS_*]*_Cart_* is the difference between the cartographic coordinates of the left camera and DGS, given respectively in Tables 5 and 7. The results are presented in Table 8.

In this case, as the DGS r.s. is leveled over the platform and its orientation matches the orientation of the platform, the linear offsets are known rigorously by means of precise measurements (see Figure 2). In this way it is possible to assess the *mean error* and the *root mean square error* of the 3 linear offset components, which were also given in Table 8. The linear accuracy of this GNSS/IMU integration can be assessed by means of the *root mean square error* of the vector T and the angular accuracy by means of the *standard deviation* of the obtained offset angles.

## 4. Discussion

The evaluation of the DGS accuracy relied on the comparison between its parameters computed using the measurements acquired in good GNSS observation conditions and the ones obtained from the exterior orientation of the left camera at the same instants, by space resection, using rigorous control points on the pavement. It was necessary first to estimate the accuracy of this exterior orientation process, which is summarized in Table 6, showing an accuracy of better than 1.5 cm in 3D positions and better than 0.1 degrees in orientation angles. This result agrees with what was expected regarding the geometric characteristics of camera/lens systems and previous experiments made during calibration tests [18]. The obtained mean linear re-projection errors of 0.35 pixels represent 0.6 cm to 1.5 cm, respectively at 4 to 10 meters distance, being that sort of distances at which the control points were observed.

The accuracy estimation of the DGS itself relied on the fact that the offset parameters between DGS and cameras must be constant. Using this condition, as shown by the results summarized in Table 8, the obtained accuracy was better than 6cm in 3D positions, and around 0.15 degrees in *roll* and *pitch* and 0.75 degrees in *heading*.

A major issue relates to the fact that the accuracy of the DGS is being assessed through an independent process of obtaining position and orientation. It can be noted, however, that the estimated accuracy with the space resection process is better than the accuracy estimated for the DGS. The relation is about 4 times better in 3D positions, 2.5 times in attitude (ω and φ angles) and 20 times in heading.

As a final remark, and regarding Table 8, if the GNSS/IMU observations made in curves are discarded, the 2nd and 3rd pairs on the table, the *heading* accuracy achieved is higher, remaining under 0.2°. As expected the heading accuracy given by the GNSS/IMU system looses quality when the vehicle is changing direction.

## 5. Conclusions

In this work the accuracy of the navigation solution provided by a DGS implemented with a particular GNSS/IMU integration was evaluated for a terrestrial platform moving in an urban environment. The used sensors were a high quality dual frequency GNSS receiver and a medium quality MEMS IMU, integrated by means of an extended Kalman filter. The results show that, for specific environments, these type of sensors can deliver good results and replace the more expensive tactical grade type of IMU.

The methodology used was to compare the DGS derived parameters with those obtained directly from the images applying space resection in the same instants. The obtained accuracy for the DGS, presented in Table 8, was of 6 cm in position parameters, 0.2° in attitude and 0.8° in heading.

## Figures and Tables

**Table 1.** Novatel manufacturer specifications.

**Novatel DL-V3 Dual Frequency GNSS Phase Receiver**	
**Horizontal Position**	**Accuracy (RMS)**
Single Point L1	1.5 m
Single Point L1/L2	1.2 m
SBAS	0.6 m
DGPS	0.4 m
RT-20	0.2 m
RT-2	1 cm + 1 ppm

**Table 2.** AHRS440 manufacturer specifications.

**Crossbow AHRS440 IMU**	
**Heading**	
Range (°)	± 180°
Accuracy (RMS)	< 1.0°
Resolution	< 0.1°
**Attitude**	
Range: Roll, Pitch	± 180°, ± 90°
Accuracy (RMS)	< 0.2°
Resolution	< 0.02°

**Table 3.** Characteristics of camera/lens used.

**Camera**	**Lens**
(3.2 × 2.4) mm^2^ CCD array	Focal Length: Vario 1.8–3.6 mm
Cell size of 5.6 μm × 5.6 μm	Iris range: F1.6 – Close
Array size of 640 × 480 pixels	Minimum Object Distance: 0.2 m
	Angle of view (Hor): 97° to 53° for a (3.2 × 2.4) mm^2^ CCD

**Table 4.** Rotations and rotation matrix conventions used for a terrestrial camera and a moving body.

**Measuring Element**	**Rotation Order**	**Rotation Matrices**
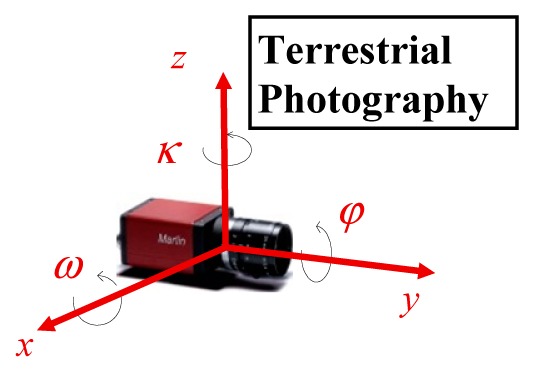	*1st - ω*	$\begin{array}{l} \text{R}={\text{R}}_{3}\left(\kappa \right)\cdot {\text{R}}_{2}\left(\varphi \right)\cdot {\text{R}}_{1}\left(\omega \right)=\\ \left(\begin{array}{ccc} \cos\kappa & \sin\kappa & 0\\ -\sin\kappa & \cos\kappa & 0\\ 0 & 0 & 1 \end{array}\right)\cdot \left(\begin{array}{ccc} \cos\varphi & 0 & -\sin\varphi \\ 0 & 1 & 0\\ \sin\varphi & 0 & \cos\varphi \end{array}\right)\cdot \left(\begin{array}{ccc} 1 & 0 & 0\\ 0 & \cos\omega & \sin\omega \\ 0 & -\sin\omega & \cos\omega \end{array}\right) \end{array}$
	*2nd - ϕ*	
	*3rd - κ*	

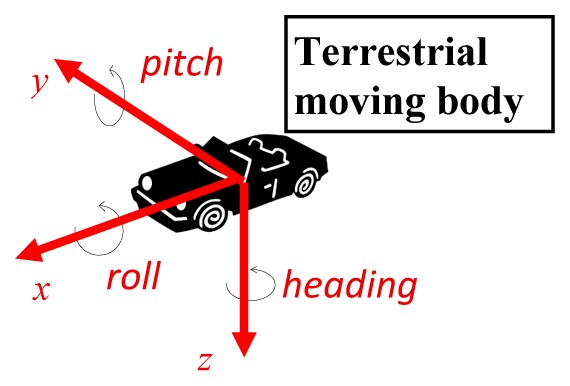	*1st - heading*	$\begin{array}{l} \text{R}={\text{R}}_{2}\left(\text{roll}\right)\cdot \text{R}1\left(\text{pitch}\right)\cdot \text{R}3\left(-\text{heading}\right)=\\ \left(\begin{array}{ccc} \cos R & 0 & -\sin R\\ 0 & 1 & 0\\ \sin R & 0 & \cos R \end{array}\right)\cdot \left(\begin{array}{ccc} 1 & 0 & 0\\ 0 & \cos P & \sin P\\ 0 & -\sin P & \cos P \end{array}\right)\cdot \left(\begin{array}{ccc} \cos H & -\sin H & 0\\ \sin H & \cos H & 0\\ 0 & 0 & 1 \end{array}\right) \end{array}$
	*2nd - pitch*	
	*3rd - roll*	

**Table 5.** Results of space resection in nine image pairs.

**Pair**	**Left Image**	**Space Resection**	**Right Image**	**Space Resection**	**Relative Orientation**

1 Straight line	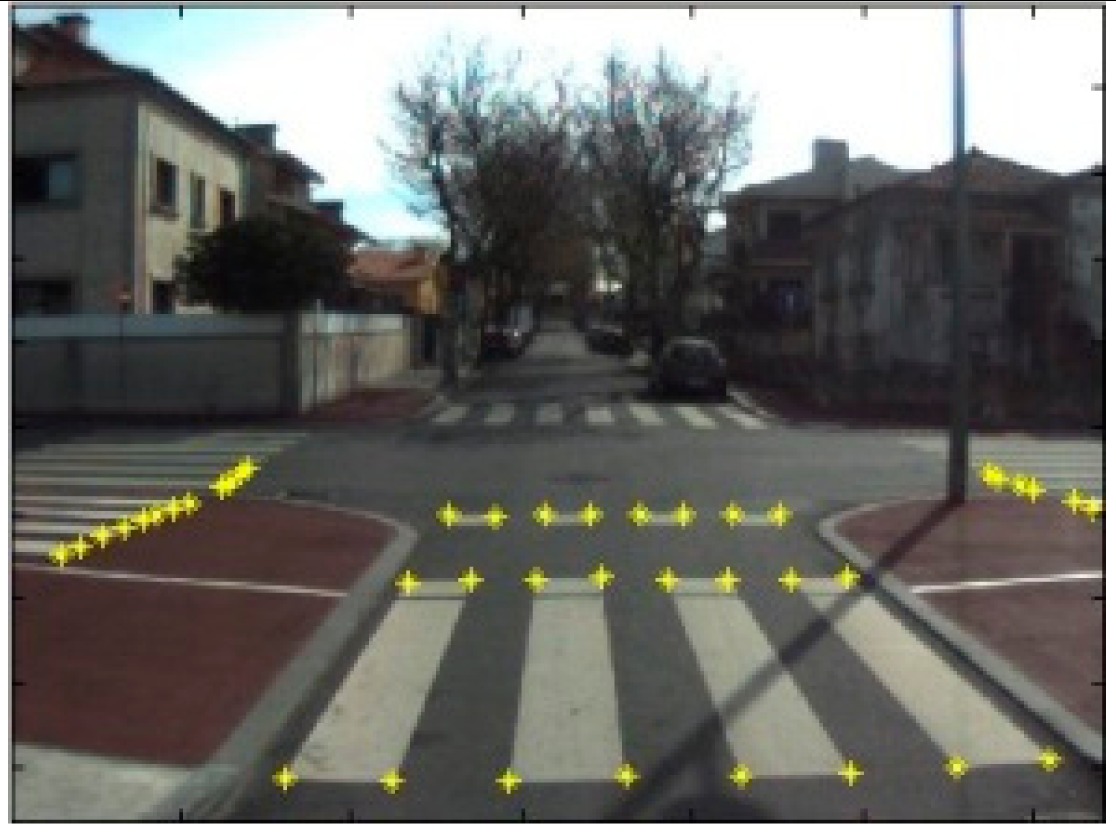	ω = −0.737°	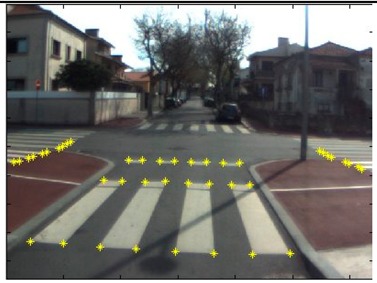	ω = −2.258°	ω_rel_ = −0.174°
		φ = 4.037°		φ = 3.858°	φ _rel_ = −1.518°
		κ = 283.200°		κ = 284.374°	κ _rel_ = 1.067°
		X = −43700.132 m		X = −43699.833m	X_rel_ = 1.043 m
		Y = 153423.686 m		Y = 153422.684m	Y_rel_ = 0.062 m
		Z = 8.435 m		Z = 8.448m	Z_rel_ = 0.021 m
		Stdxy = 1.3;1.3 pix		Stdxy=1.7;1.5 pix	T = 1.045 m

2 Curve	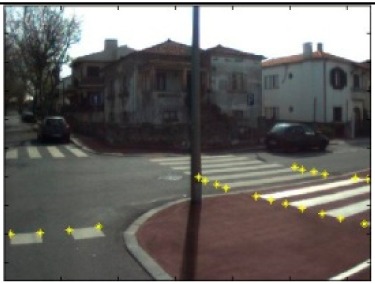	ω = 1.777°	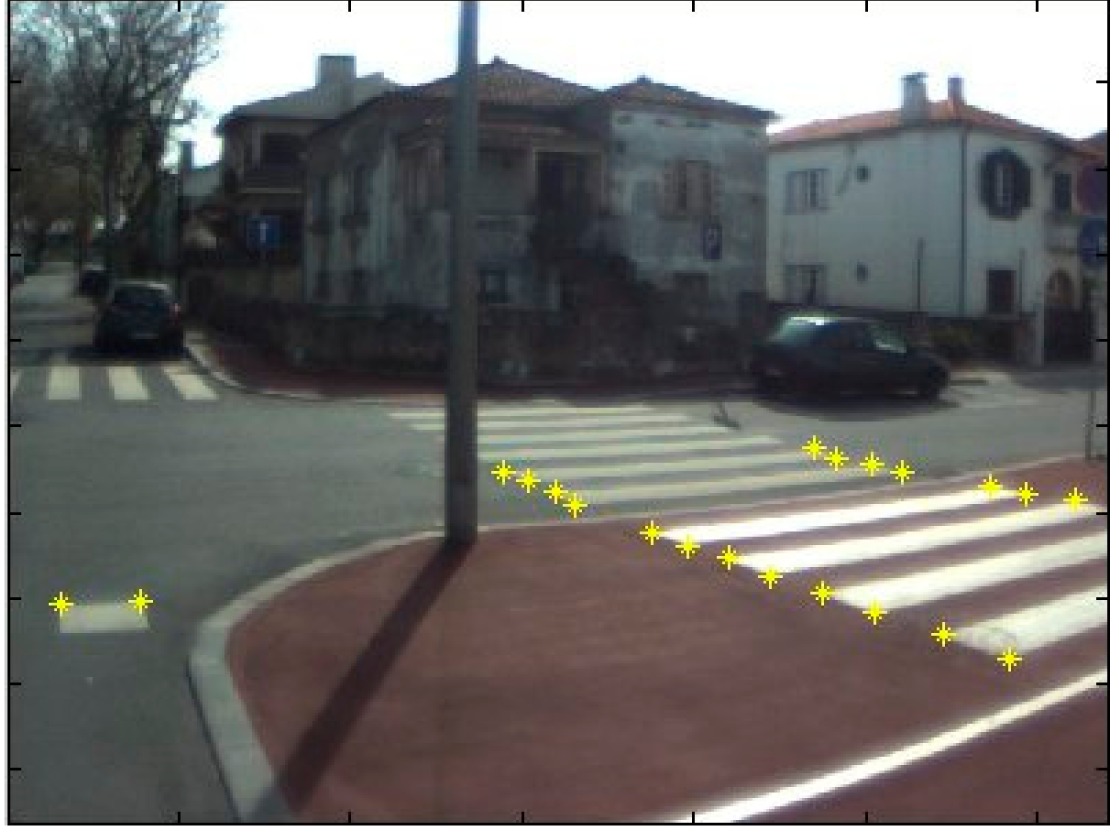	ω = − 0.397°	ω _rel_ = −0.147°
		φ = 4.199°		φ = 4.706°	φ _rel_ = −1.459°
		κ = 255.460°		κ = 256.671°	κ _rel_ = 1.102°
		X = −43694.735 m		X = −43694.946 m	X_rel_ = 1.035 m
		Y = 153423.886 m		Y = 153422.871 m	Y_rel_ = 0.048 m Z_rel_
		Z = 8.423 m		Z = 8.431m	= 0.024 m
		Stdxy = 2.4;1.2 pix		Stdxy = 2.2;0.9 pix	T = 1.037 m

3 Curve	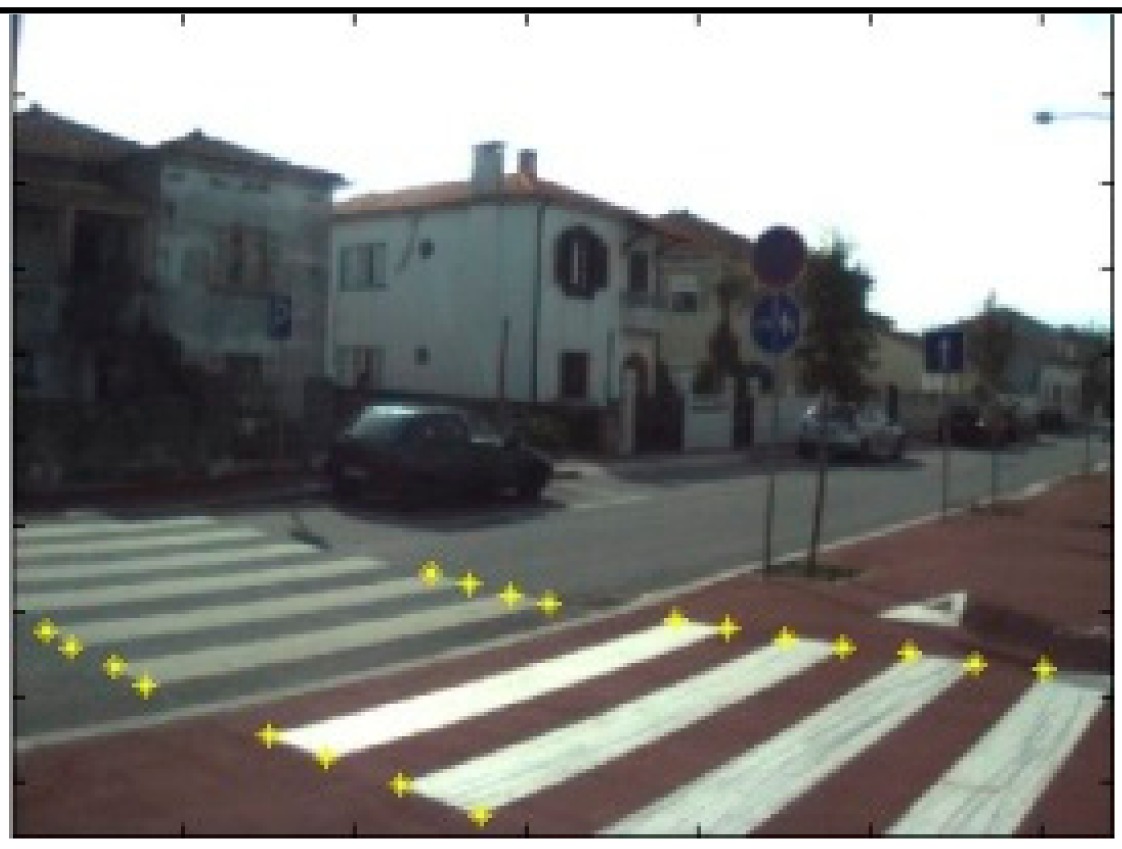	ω = 0.365°	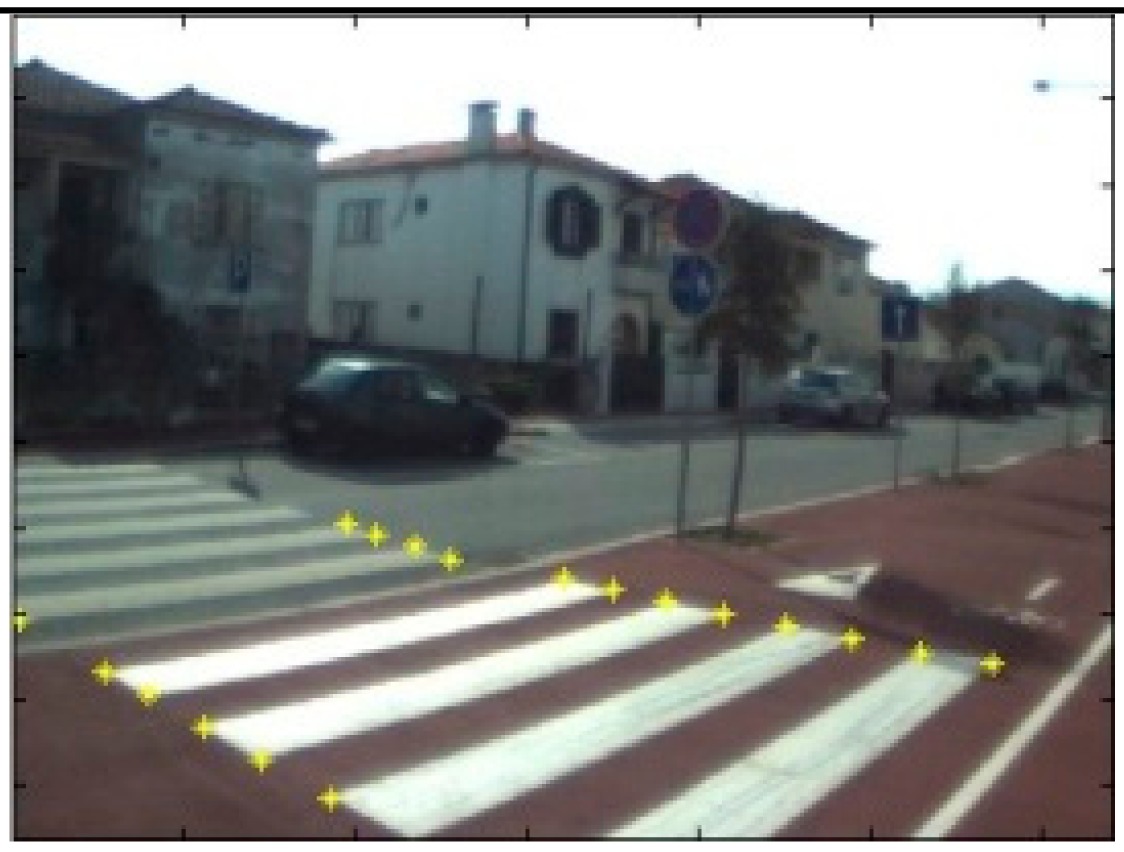	ω = −0.753°	ω _rel_ = −0.224°
		φ = 3.092°		φ = 4.259°	φ _rel_ = −1.599°
		κ = 231.655°		κ = 232.757°	κ _rel_ = 1.028°
		X = −43692.415 m		X = −43692.993 m	X_rel_ = 1.038 m
		Y = 153422.506 m		Y = 153421.642 m	Y_rel_ = 0.081 m
		Z = 8.483m		Z = 8.530 m	Z_rel_ = 0.021 m
		Stdxy = 1.7;1.3 pix		Stdxy = 1.3;1.2 pix	T = 1.041 m

4 Straight line	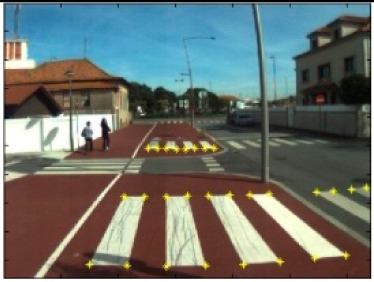	ω = −4.790°	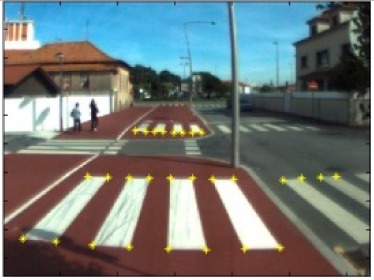	ω = −4.626°	ω _rel_ = −1.121°
		φ = −0.199°		φ = −1.677°	φ _rel_ = −1.483°
		κ = 10.994°		κ = 12.053°	κ _rel_ = 1.054°
		X = −43687.679 m		X = −43686.669 m	X_rel_ = 1.042 m
		Y = 153411.606 m		Y = 153411.868 m	Y_rel_ = 0.064 m
		Z = 8.576 m		Z = 8.570 m	Z_rel_ = 0.013 m
		Stdxy = 1.4;0.6 pix		Stdxy = 1.3;0.9 pix	T = 1.044 m

5 Straight line	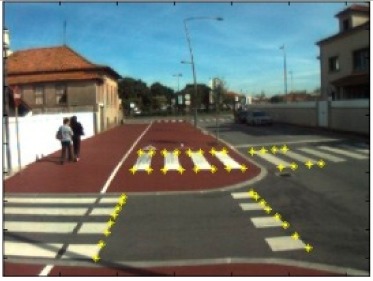	ω = −4.645°	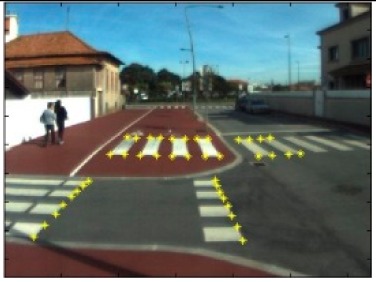	ω = −4.460°	ω _rel_ = −0.096°
		φ = 0.253°		φ = −1.218°	φ _rel_ = −1.479°
		κ = 10.846°		κ = 11.866°	κ _rel_ = 1.017°
		X = −43689.485 m		X = −43688.495 m	X_rel_ = 1.020 m
		Y = 153418.980 m		Y = 153419.236 m	Y_rel_ = 0.065 m
		Z = 8.487 m		Z = 8.480 m	Z_rel_ = 0.018 m
		Stdxy = 1.3;1.1 pix		Stdxy = 1.1;1.0 pix	T = 1.023 m

6 Straight line	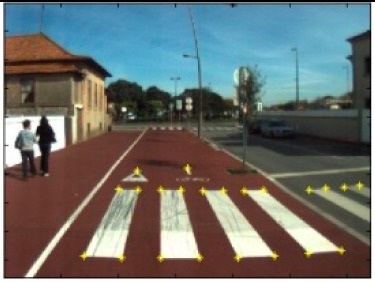	ω = −3.787°	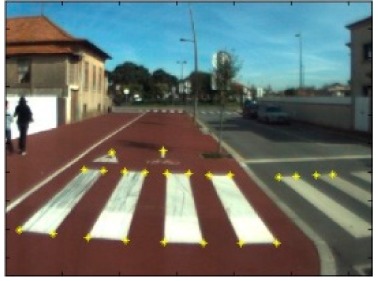	ω = −3.642°	ω _rel_ = −0.140°
		φ = −0.965°		φ = −2.585°	φ _rel_ = −1.621°
		κ = 10.027°		κ = 11.028°	κ _rel_ = 0.995°
		X = −43691.099 m		X = −43690.102 m	X_rel_ = 1.026 m
		Y = 153425.459 m		Y = 153425.712 m	Y_rel_ = 0.075 m
		Z = 8.513 m		Z = 8.526m	Z_rel_ = 0.013 m
		Stdxy = 1.1;1.0 pix		Stdxy = 1.5;1.5 pix	T = 1.029 m

7 Straight line	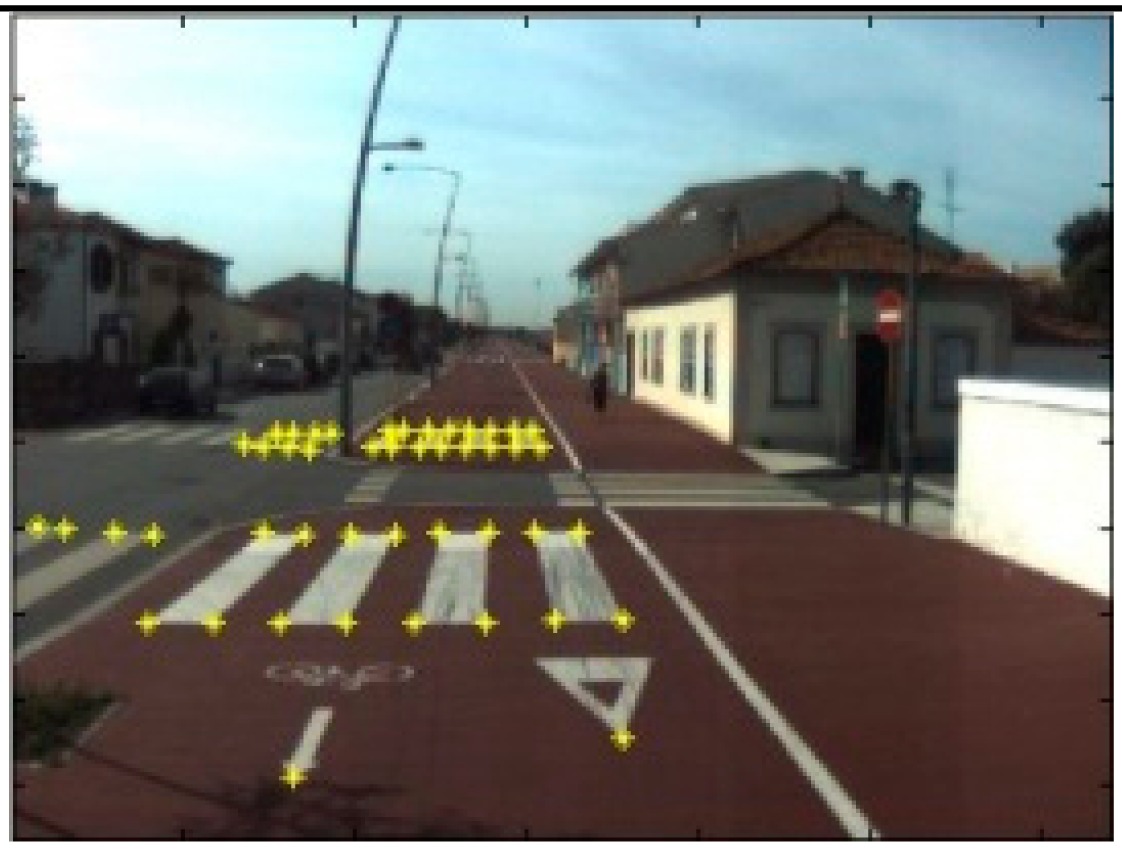	ω = 4.873°	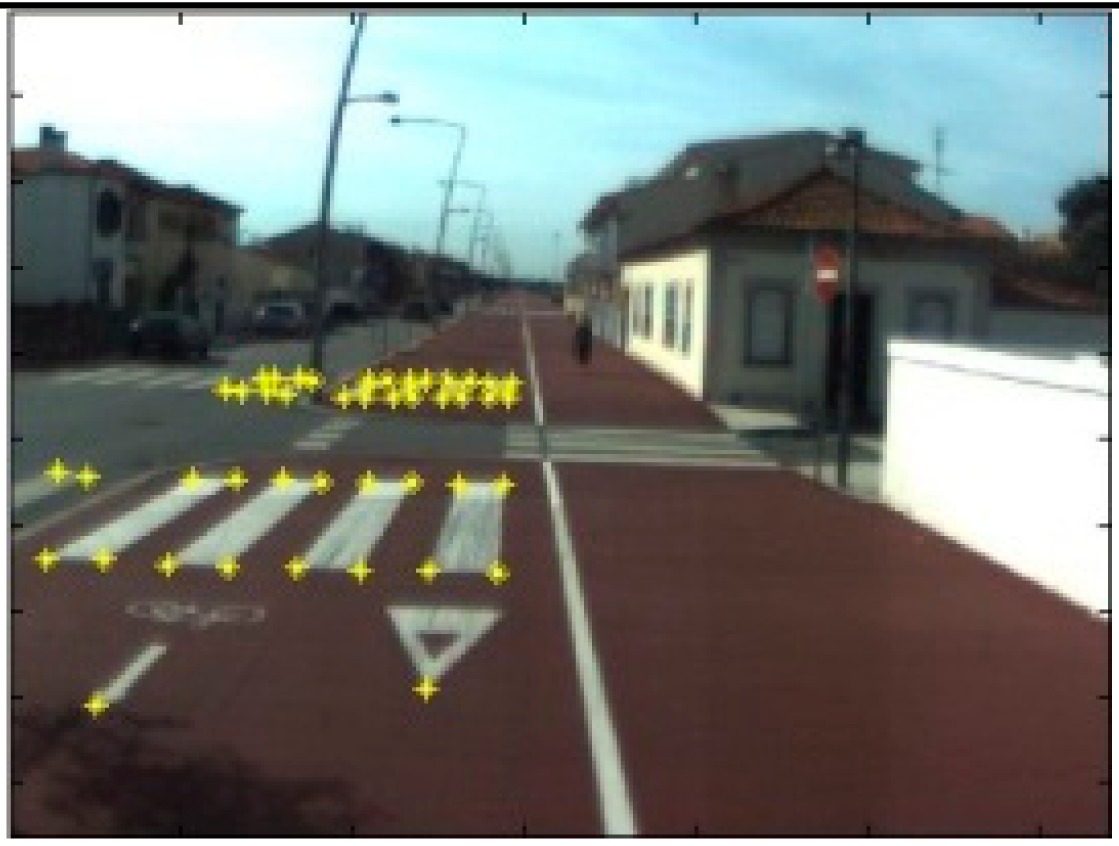	ω = 4.798°	ω _rel_ = −0.181°
		φ = 1.225°		φ = 2.613°	φ _rel_ = −1.379°
		κ = 190.571°		κ = 191.616°	κ _rel_ = 1.041°
		X = −43694.993 m		X = −43696.017 m	X_rel_ = 1.047 m
		Y = 153440.921 m		Y = 153440.706 m	Y_rel_ = 0.019 m
		Z = 8.838 m		Z = 8.874 m	Z_rel_ = 0.032 m
		Stdxy = 1.6;0.8 pix		Stdxy = 1.9;0.9 pix	T = 1.047 m

8 Straight line	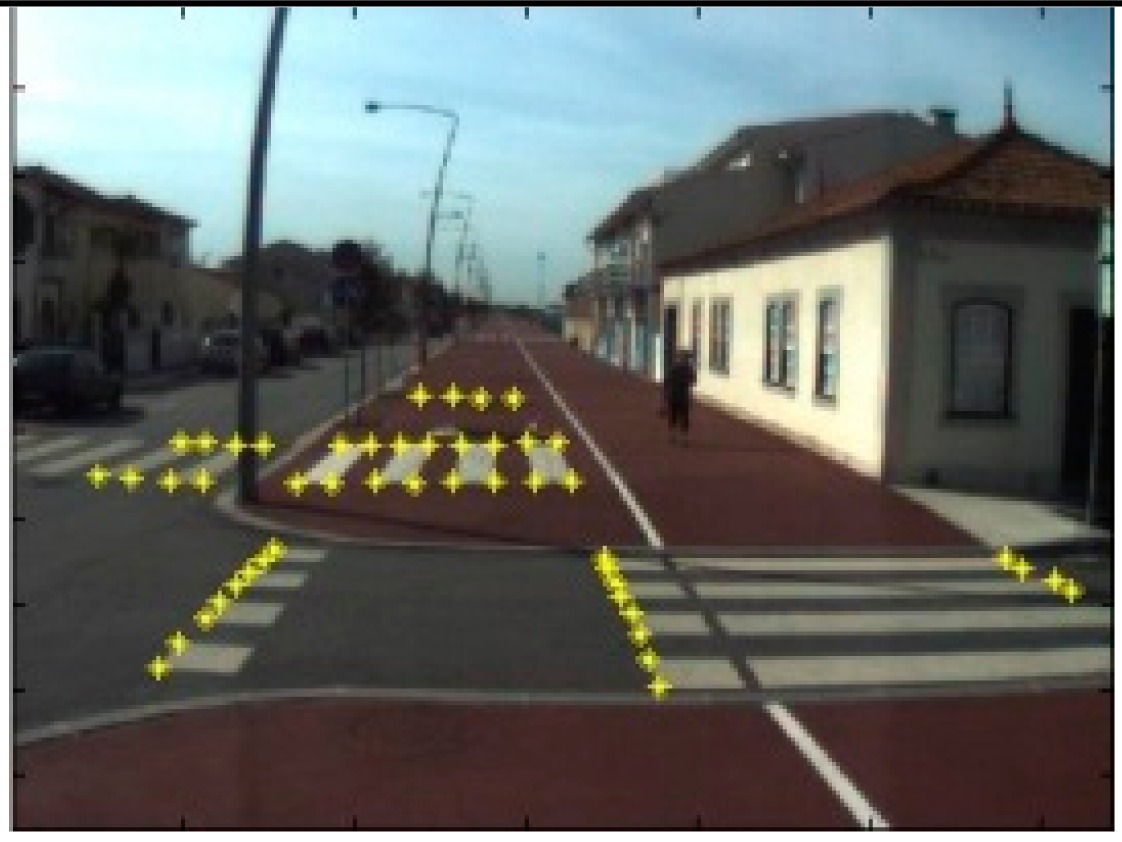	ω = 5.271°	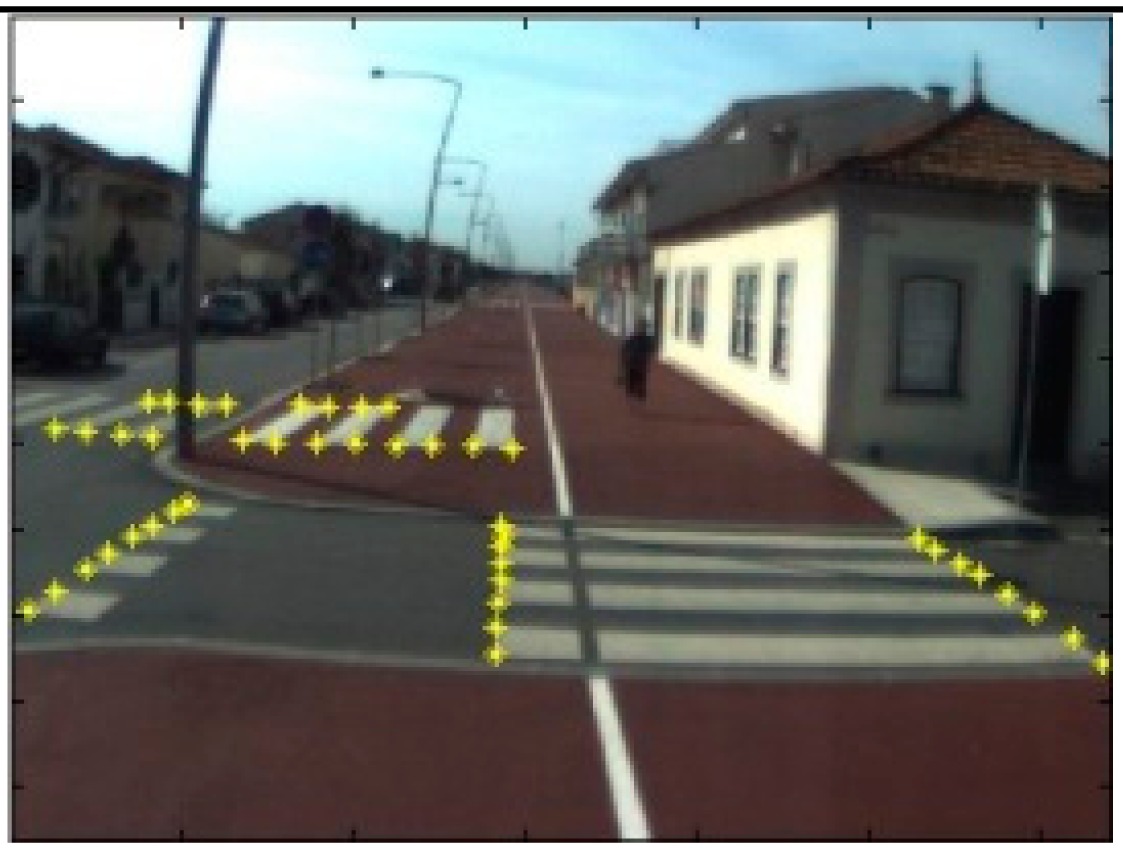	ω = 5.139°	ω _rel_ = −0.154°
		φ = 2.060°		φ = 3.609°	φ _rel_ = −1.547°
		κ = 190.560°		κ = 191.576°	κ _rel_ = 1.008°
		X = −43692.895 m		X = −43693.894 m	X_rel_ = 1.033 m
		Y = 153432.684 m		Y = 153432.413 m	Y_rel_ = 0.079 m
		Z = 8.678m		Z = 8.708 m	Z_rel_ = 0.018 m
		Stdxy = 1.9;1.0 pix		Stdxy = 2.1;1.1 pix	T = 1.036 m

9 Straight line	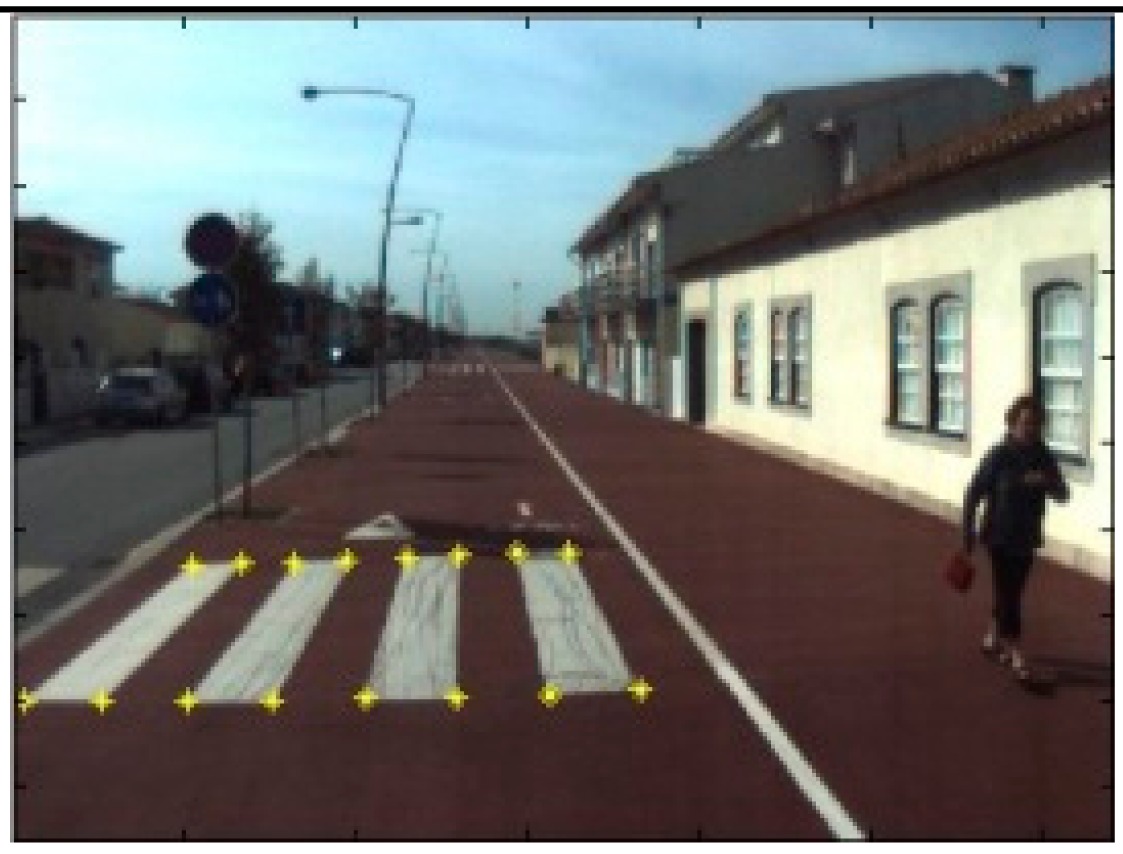	ω = 4.823°	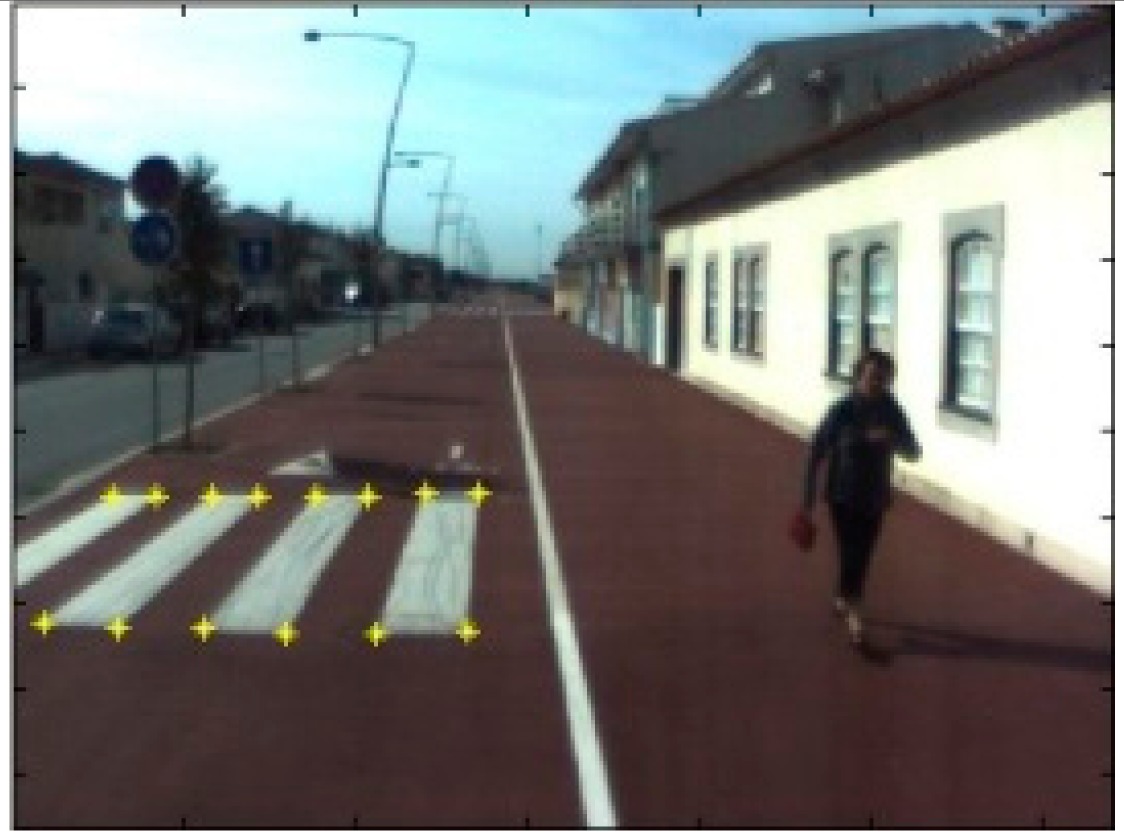	ω = − 4.776°	ω _rel_ = −0.175°
		φ = −0.074°		φ = 1.269°	φ _rel_ = −1.333°
		κ = 189.460°		κ = 190.526°	κ _rel_ = 1.063°
		X = −43691.246 m		X = −43692.288 m	X_rel_ = 1.064 m
		Y = 153425.281 m		Y = 153425.062 m	Y_rel_ = 0.044 m
		Z = 8.536 m		Z = 8.540m	Z_rel_ = 0.023 m
		Stdxy = 1.5;1.0 pix		Stdxy = 1.3;0.7 pix	T = 1.065 m

**Table 6.** Accuracy estimation of the cameras' exterior orientation by space resection.

	***Δω***	***Δ φ***	***Δ κ***	***Δx (m)***	***Δy (m)***	***Δz (m)***	***ΔT(m)***
*Std*	0.037°	0.094°	0.034°	0.013	0.020	0.006	0.012
*Mean E*							−0.003
*RMSE*							0.012

**Table 7.** Position and orientation parameters obtained from GNSS/IMU processing in the same instants of the photogrammetric pairs.

**Pair**	**X (m)**	**Y (m)**	**H (m)**	**Roll (°)**	**Pitch (°)**	**Heading (°)**
*1*	−43,700.51	153,422.84	8.38	1.848	0.457	75.039
*2*	−43,695.31	153,422.95	8.41	0.829	0.417	101.501
*3*	−43,693.06	153,421.66	8.44	0.711	3.761	125.160
*4*	−43,687.48	153,411.23	8.57	−0.039	−0.849	347.533
*5*	−43,689.25	153,418.56	8.52	−0.498	−0.721	347.775
*6*	−43,690.85	153,425.03	8.51	0.954	−1.791	348.608
*7*	−43,695.77	153,440.53	8.84	−1.404	0.863	168.090
*8*	−43,693.66	153,432.23	8.70	−2.073	0.330	168.016
*9*	−43,692.01	153,424.82	8.48	−0.092	1.061	169.328

**Table 8.** Offset parameters of left camera in GNSS/INS reference system.

**Pair/offsets**	**Offset X (m)**	**Offset Y (m)**	**Offset H (m)**	**T vector (m)**	**Offset Roll (°)**	**Offset Pitch (°)**	**Offset Heading (°)**
*1*	−0.411	0.188	0.061	0.456	1.075	−5.792	1.697
*2*	−0.579	0.158	0.018	0.601	0.867	−5.416	2.927
*3*	−0.558	0.024	0.073	0.563	0.885	−5.540	3.010
*4*	−0.497	0.085	0.011	0.505	0.845	−5.580	1.423
*5*	−0.524	0.133	−0.033	0.541	0.770	−5.316	1.372
*6*	−0.537	0.138	0.016	0.555	0.967	−5.493	1.256
*7*	−0.460	0.106	0.014	0.472	1.239	−5.602	1.168
*8*	−0.462	0.043	0.000	0.464	1.051	−5.498	1.184
*9*	−0.462	0.027	0.063	0.467	1.135	−5.777	1.155
*Mean*	−0.499	0.100	0.025	0.514	0.982	−5.557	1.688
*Std*	0.055	0.059	0.034	0.053	0.154	0.155	0.746
***Known Values***	**−0.522**	**0.125**	**0.020**	**0.537**			
*Mean Error*	0.023	−0.025	0.005	−0.023			
*RMSE*	0.057	0.061	0.033	0.055			
